# Proposal: Recommendation on Measuring and Providing Mass Spectra as Chemical Information of Organic Molecules (Secondary Publication)

**DOI:** 10.5702/massspectrometry.A0076

**Published:** 2019-10-25

**Authors:** MassBank Database Committee, The Mass Spectrometry Society of Japan

## INTRODUCTION

Many of us have surely had the experience of measuring the mass spectrum of a substance with a specific purpose in mind and then having struggled to ascertain as to what should be considered important during the analysis, and which mass spectrum should be used to represent the substance. The MassBank Database Committee of the Mass Spectrometry Society of Japan has been holding discussions since the summer of 2017 to propose a set of guidelines to answer such questions. On request, several members of the society have provided us with their opinions on these questions.

This paper aims to provide specific guidelines, based on our discussions and society member’s opinions, on the methods of measurement and the correct manner of representation of: (1) mass spectra with electron ionization (EI), which is widely used in the mass spectrometry of substances, and (2) product ion spectra, obtained by the combination of electrospray ionization (ESI), which is a soft ionization, and tandem mass spectrometry (MS/MS), which analyzes product ions by a high resolving mass analyzer. We assume situations where researchers, technicians, undergraduate and graduate students measure mass spectra and submit them as experimental data that lead to conclusions in academic papers, or as supporting data for documentation. In order to provide unambiguous and reliable guidelines that incorporate the latest developments in the field of mass spectrometry, we request the society members to submit their opinions to this committee (Mail address: msdb@mssj.jp).

## GENERAL PLAN OF THE PROPOSAL

In Section 1, we propose methods to accurately measure the *m*/*z* and quantity of ions, and thereby create metadata to support them. Here, metadata are used as records of “Materials and Methods” related to mass spectrum acquisition. In Section 2, we propose guidelines to measure and use product ion spectra as representatives of data aiding the analysis of molecular structure. We suppose that the substance analyzed here is an organic molecule with a molecular weight up to 1,500 Da. Section 3 is a collection of the opinions that some society members, who are specialists of specific topics of mass spectrometry, sent to the authors in response to our questions.

We wish to refer to the following books and terminology lists to develop an understanding of the principles of mass spectrometry.

• “Mass Spectrometry. A Textbook, Japanese version” (J. H. Gross, translated by the Publishing Committee of The Mass Spectrometry Society of Japan, Maruzen Shuppan, 2017)
• “Current Mass Spectrometry” (edited by Mitsuo Takayama, Shigeo Hayakawa, Yoshihiko Takinami, and Yoshinao Wada, Kagaku Dojin, 2013)
• “Mass Spectrometry—Fundamentals” (written and edited by Michisato Toyoda, Kokusai Bunken, 2016)
• “Mass Spectrometry related terminology—Version 3” (Terminology Committee of The Mass Spectrometry Society of Japan, WWW version, 2009) URL http://www.mssj.jp/publications/pdf/MS_Terms_2009.pdf

## 1. THE BASICS: MEASURING *m*/*z* AS PHYSICAL DATA

Mass spectrometry (MS) is a technique that measures the *m*/*z* and abundance of ions in the gas phase. A mass spectrum is a two-dimensional representation of ions with the measured *m*/*z* on the *x*-axis and the abundance on the *y*-axis. In order to measure *m*/*z* as a physical data of the substance, it is very important for the *m*/*z* values to be reproducible. A mass spectrum with good reproducibility is obtained by using a correctly calibrated instrument (Section 3.1) and taking measurements under appropriate analytical conditions.

Such spectra should:
  • give the peak profile that is symmetrically shaped and has the centroid on the peak top when measured by profile acquisition mode (Section 3.2);
  • show the *m*/*z* value of the easily attributable molecular ions such as M^+^* and [M+H]^+(1)^ with the accuracy comparable or better than guaranteed by the instrument manufacturers.^(2)^

Such reproducibility can be secured by keeping record of the calibration method and the measurement conditions.^(3)^ It would be ideal to show the standard deviation σ, which is the indicator of accuracy, for the measured *m*/*z* values.

## 2. PURPOSE OF MEASUREMENT: USE OF MS TO ANALYZE SUBSTANCES

### 2.1 Measurement of mass spectrum to determine the elemental composition of a substance

Mass spectrometry is the most suitable analytical method for the determination of elemental composition, which is obtained by measuring the *m*/*z* of the molecular ion with a mass analyzer of high mass resolving power. However, it is not easy to identify the molecular ion on a mass spectrum. The mass spectra measured using the electron ionization (EI-MS) show M^+^* as well as the fragment ions formed as a result of the dissociation of M^+^*. The terms “product ion” and “dissociation” are preferred to “fragment ion” and “fragmentation” hereafter. In some substances, their M^+^* easily dissociate and are never observed in the spectra. Therefore, it no longer remains possible to assign the peak of the largest *m*/*z* to M^+^*.

In order to observe the molecular ion, a soft ionization method,^(4)^ such as ESI-MS, is used to generate stable molecular ions without significant dissociation. At times, you might observe that the substance forms adduct ions with alkali ions like Na^+^, K^+^ and Li^+^ in addition to the molecular ion [M+H]^+^ (Section 3.3). When a molecular ion is eventually identified, the elemental composition that is calculated as the best fit to the observed *m*/*z* value, is listed along with such factors as the measurement accuracy, observation of stable characteristic halogen isotope peaks, the nitrogen rule, *etc*. If one needs to choose from multiple candidates of the elemental composition, the composition also satisfying those of all the product ions observed should be selected (Section 2.2.2).

### 2.2 Measuring mass spectrum as the data that analyzes the structure of substances

When analyzing the structure of a substance, we must remember that mass spectrometry provides the information of the interatomic connectivity, but none of the stereochemistry. To identify a substance by using mass spectrometry means to determine the atoms, which the substance is composed of, and their connectivity.

The primary structure of a chemical substance is built by covalent bonds. The chemical energy, or in other words, stability of each covalent bond depends not only on the two atoms that pairwise form the bond, but also on their neighboring atoms and resonance structures. In case of the molecular ion, the effect of the localized charge on the chemical energy is more pronounced. Such differences in the chemical energies are observed as those in the *m*/*z* and intensity of the product ion peaks that are generated by different dissociation pathways of the molecular ion.

Structure analysis using mass spectrometry requires some prior knowledge of the substance structure. Product ion spectra of the substance are examined and interpreted whether observed product ions are consistently attributable to specific parts of the structure. Thus the structure analysis approves the known or predicted structure and even proposes more exact structure. To conduct such an analysis, we refer to “known information,” such as mass spectra libraries, empirical rules of dissociation, and literatures that have analyzed similar substances using mass spectrometry and other analytical methods.

To verify the correctness of the proposed structure, one must select to show one or more product ion spectra. When making these choices, it is necessary to evaluate the importance and reliability of the attribution by considering the original purpose of the structure analysis and the quality and quantity of the known information. An explanation of this evaluation should be attached with the mass spectra.

#### 2.2.1 Identifying or elucidating structure using EI-MS

As the analytical conditions have been standardized for the EI-MS method, usually using electronic energies of 70 eV for ionization, we are able to obtain reproducible mass spectra. Taking advantage of this reproducibility, spectral libraries can be used to identify sample substances. As an example of such a library, NIST/EPA/NIH Mass Spectral Library (NIST Library) is a collection of mass spectra of standard substances analyzed by EI-MS and gas chromatography-MS (GC-MS). Using the spectrum of a sample as a query, we search for a match from the spectra available in the library. If both the elemental composition of M^+^* and the mass spectrum match, this indicates that the sample and the standard substance have the same atomic connectivity.

In order to determine that they are one and the same compound,^(5)^ it would be preferable to measure other physicochemical properties, like the following, of the sample substance and match them with literature values for the same properties of the standard substance;

• Elemental compositions (Section 2.1).
• Other physicochemical properties such as retention time on GC.

Property value compilations are available on databases for chemical compounds (PubChem, *etc.*).

Metadata describing the methods of mass spectrometry analyzing the molecular ion in details should be attached to the values of the *m*/*z* and elemental composition of the molecular ion of the sample substance. The spectrum of the sample substance, the record ID of the matched mass spectrum of the standard substance in the library, and the experimental data from the physical property evaluations of the sample substance that show agreement with the literature values, should be presented.

If a mass spectrum is very similar but does not match any of the spectra in the library,^(6)^ one should investigate the following possibilities.

• The sample substance is similar in the structure to these standard substances in the library.

The difference in the elemental composition between the sample and the standard substances is helpful to elucidate the structure of the sample substance. Then, one should measure the physicochemical properties of the sample substance and compare the measured properties with those of the standard substance having the similar structure, if reported on the literatures. If the substance having the elucidated structure is available as a reagent, one could purchase it or, if not, synthesize it. The substance, purchased or synthesized, and the sample substance should then be separately analyzed by EI-MS to verify that the mass spectra for both match. The observed *m*/*z* and elemental composition of the molecular ions on the both mass spectra should be reported, as well as the metadata from measuring the molecular ion should be attached. Furthermore, mass spectra of both the sample substance and the purchased or synthesized substance should be presented together with the experimental data, showing that the physical property values for the sample substance are in agreement with literature values.

#### 2.2.2 Identifying or elucidating the structure using a soft ionization method (MS/MS method)

Here, we discuss the MS/MS method that uses ESI as a typical example of the soft ionization. Product ion spectra are obtained by the first mass spectrometry selecting the molecular ion generated by ESI as the precursor ion, then through the collision-induced dissociation (CID) of the precursor ion giving the product ions, followed by the second mass spectrometry analyzing the product ions. MS/MS can be measured by varying several analytical conditions, such as the magnitude of collision energy for the dissociation of the precursor ion, *i.e.*, whether to use low- or high-energy CID, which types of ion analyzers are combined to a hybrid-type mass spectrometer, *etc*. In other words, there are many factors that can affect the reproducibility of product ion spectra here. As a result, no standard analytical conditions have not been specified for the MS/MS method. It has been said that the MS/MS method has low reproducibility as the obtained product ion spectrum varies with different analytical conditions, even for the same substance.

With the progress of the study on the mechanisms responsible for the differences in the product ion spectra under different analytical conditions, it has become possible to obtain spectra with relatively high possibilities of the reproducibility (Sections 3.5, 3.6). It could apply these differences in mechanisms to control the bond dissociations and rearrangement reactions in the molecular ion in order to obtain detailed structure information.

A good understanding of the CID reaction mechanism, the characteristics of dissociation dependent on the analyzer type, and the measurement method of product ion spectrum, will prove useful for optimizing the analytical conditions as well as for the structure analysis .

In MS/MS measurements that use soft ionization method, it is essential that detailed metadata are documented in order to reproduce the product ion spectrum and carry out the structure analysis that attributes product ions to the substance structure.

At this stage, it is relatively easy to assign the elemental composition as a chemical annotation to the product ions obtained from the measurements. The assignment confirms the elemental composition of the molecular ion predicted in Section 2.1. In addition, it could provide the experimental data that determine the accuracy and reliability of the *m*/*z* measurements in the product ion spectra, and that prove the presence or absence of contamination. Finally, when the structure of the analyzed substance is elucidated, one can attribute with high reliability as to which covalent bonds of the structure have been cleaved to form the product ions.

#### 2.2.3 Known information for structure analysis using product ion spectra

The libraries of mass spectra of standard substances analyzed by EI-MS was intended for the identification of substances (Section 2.2.1). Libraries of product ion spectra are also available for the same purpose, but limited to the lipid identification so far (Section 3.7). For other substances, the analytical conditions of the MS/MS method could not be standardized yet. Therefore, the purpose and usefulness of the libraries of product ion spectra, such as MassBank, become evident when they are referenced for purposes such as obtaining optimal analytical conditions, performing structure analysis or crosschecking the deduction made through such analysis, rather than for the explicit identification of substances (Section 3.8). When using these libraries, it is necessary to pay attention to the following:

• Even if the CID conditions are the same, product ion spectra vary to a large extent depending on the type of instruments used and it is therefore necessary to proceed with caution. For example, when comparing a product ion spectrum measured by using “High-energy CID on magnetic sector and time-of-flight/time-of-flight (TOF/TOF),” “quadrupole-TOF (Q-TOF) and triple quadrupole mass analyzer (QqQ),” and “Ion-trap including Fourier transform ion cyclotron resonance (FT-ICR) and Orbitrap,” we recommend referring to the mass spectra measured by using the same type of instruments.
• The product ion spectrum is observed as a result of the dissociation of the molecular ion. It is necessary to be careful that even when measuring the mass spectra of the same substance, different molecular ions, such as [M+H]^+^, [M+Na]^+^, and [M−H]^−^, can produce different product ion spectra for each.

In addition to MassBank, the website Metabolomics.jp (http://metabolomics.jp/wiki/Index:MassBank) is available as a library which attributes each product ion to a specific substructure of the molecular ions. Searching the library with the molecular formula of the product ion as a query provides possible structures of the molecular ions that commonly give the query product ion. This is useful for finding real examples to the question, “Have these bonds really been cleaved?”

Recent advances in information science applied to mass spectrometry data, which is called “MS informatics,” are remarkable. Using an informatics tool to elucidate the structure of molecular ions may be useful for structure estimation (Section 3.9) although it may not form a part of the conventional “known information.”

#### 2.2.4 Which product ion spectrum should be submitted as evidence data?

Regardless of the analytical conditions and mass resolving power of instrument, the measured product ion spectra will exhibit the structure features of the molecular ion, observed under these specific conditions, as long as they have been measured correctly using the MS/MS method. One cannot deny the fact that two spectra, one in which a large number of product ions are observed and the other in which only a few are observed, give some information on the structure of the substance. The number of product ions observed in a product ion spectrum is irrelevant to the quality of the spectra. Therefore, when attaching mass spectra as the evidence data of the structure analysis of the sample substance to a documentation, we recommend reporting the *m*/*z* value of the precursor ion and its supporting spectrum observing the molecular ion, and selecting product ion spectra observing both the product ions and the precursor ion (Section 3.6), along with their metadata.

### 2.3 Submitting product ion spectra of a known substance to MassBank

One should take measurements of MS/MS after the structure of the substance is confirmed. If this substance is a mixture of stereoisomers or geometrical isomers, the isomer ratios should be accurately recorded in the metadata, even if it is known to give no effect on mass spectra. It is necessary to keep in mind that as long as the MS/MS measurement has been taken properly, the product ion spectra invariably represents some characteristics of the structure of the substance under study, regardless of the analytical conditions used for the measurement. When performing MS/MS of any substances of known and identified structures, it is recommended to submit the *m*/*z* values of the precursor ion and the metadata, all product ion spectra with their metadata, to MassBank. The MassBank Database Committee is supporting the disclosure procedures, and it is possible to consult them in advance by writing to ‹msdb@mssj.jp› (Section 3.10).

## 3. OPINIONS TO THE GUIDELINES SUBMITTED BY SOCIETY MEMBERS

### 3.1 Mass calibration

One should adjust the ion source and the mass analyzer according to the recommendations of the instrument manufacturers and adjust the mass resolution to suit the measurement purpose. They recommend external mass calibration using a standard substance every day at the beginning and end of work, and also before and after each measurement, as necessary. Internal mass calibration is used during measurement of the product ion spectrum of the substance. It is a method that calibrates the mass using the *m*/*z* value of several product ions that can be attributed with certainty. This method is known to provide satisfactory results with considerable accuracy when measuring with the TOF mass analyzer which is high mass resolving power. A calibration method is deemed acceptable if the details of the product ions used as well as those of the calibration method are recorded and provided in the metadata.

### 3.2 Shape of the peak profile

This description assumes that a peak profile follows a Gaussian distribution. Even if the profile is symmetrical and has two peaks on the left and right side of the center, the centroid of the profile may not necessarily give any physically meaningful value, *m*/*z*. The reflectron type TOF mass analyzer is known to be particularly prone to tailing if the peaks are narrow. The questions which arise when there is tailing are whether it is even meaningful to evaluate the centroid, whether the peak top value has any physical meaning, and whether the centroid of the upper half of the peak holds any physical significance. One wonders if there is no way to make this work. For this, it is necessary to thoroughly respond to each of the issues mentioned above. An acceptable shape of the peak does not necessarily indicate high accuracy, and we must also decide the accuracy which we wish to discuss in this context.

The peak shape is also affected by the sample concentration. Mass spectra should be measured using an optimum sample concentration. If the concentration is too low, the spectrum will be affected by background noise. If it is too high, one may encounter problems like the profile becoming asymmetrical and the measurement of the peak intensity giving an incorrect value. A sample concentration should be chosen so that the ion quantity is within the appropriate response range of the detector where the signal intensity increases linearly with the ion quantity.

The scan speed may also affect the peak shape. On GC-MS, the elution peak of a substance may be very narrow. When performing the analysis using a quadrupole analyzer, the scan speed needs to be adjusted suitably to measure the *m*/*z* value at the center of the elution peak.

### 3.3 Contamination and noise

It is important to pay attention to the maintenance of analytical instruments used for GC-MS and liquid chromatography-MS (LC-MS). We recommend that system performance checks are carried out regularly. Contamination-affected peaks and alkali metal-added (Na^+^, K^+^, Li^+^, *etc.*) molecules are often observed as a result of the dirt on the injection port, septum, or the column tip, as well as from deterioration of the column and contamination of the ion source.

As metal-added molecules are less prone to dissociate compared to [M+H]^+^, they are unsuitable precursor ion for the MS/MS measurements intended for structure analysis (Section 2.2.2). Even when [M+H]^+^ is chosen as the precursor ion, generation of metal-added ions effectively reduces the quantity of [M+H]^+^ by a considerable amount. Such a reduction is counterproductive when the sample concentration is as low as near the limit of detection.

When introducing the sample into the MS instrument, it should be either introduced directly or after an LC separation, according to the situation. As erasing contamination-derived peaks from mass spectra is equivalent to falsification of experimental data, it must be avoided at all costs. If it is obvious that a peak has arisen as a result of contamination, it should be annotated. Common contaminations have been compiled in the following review.

B.O. Keller *et al.*, *Anal. Chim. Acta*, 627, 71–81 (2008). doi: 10.16/j.aca.2008.04.043

It is recommended not to calculate a background-subtracted differential spectrum or to increase the peak intensity detection threshold when there is significant noise. Noise should be controlled through instrument maintenance.

### 3.4 Use of spectral libraries: Part 1

• Although the dissociation of a particular molecular ion generates a fixed set of product ion species and their relative quantities, the reverse does not hold true. The same set of product ion species could generate multiple molecular ion species having different bonding relationships, *i.e.*, molecular structures. For example, if three molecular ion species can be predicted from a single product ion spectrum, the probability of predicting the correct structure for the ion under study is only 0.33.
• Since the mass spectra collected in the NIST library are provided as integer masses (*m*/*z* values are given as integers), even if two *m*/*z* values match, their elemental compositions can differ (*i.e.*, they are chemically unrelated to one another).
• The NIST library is incomplete. The library, 2017 version, is a collection of 310,000 mass spectra obtained from the analysis of approximately 262,000 compounds using the EI-MS method. In contrast, the PubChem (as of October 2017) database of chemical compounds has a collection of approximately 94 million compounds, each with less than 1,000 atoms and bonds, combined. The NIST library thus covers only 0.28% of the known chemical compounds. Even if we consider the fact that stereoisomers give the same mass spectra and limit our consideration to only volatile substances, the coverage may rise only by a few percent. The inability to find an exact match in the library for a particular spectrum under study is primarily due to this incompleteness.

### 3.5 Selection of precursor ion

Whether or not the correct precursor ion has been selected, is an important factor that determines the “sample purity” of the product ion spectrum. The mass resolution and the accuracy of the mass calibration used for this selection and the setting of the “window” width used for the selection are all related to each other. The window width varies significantly depending on the mass analyzer type and the conditions set for the measurement. This can vary starting from several Daltons or more when using quadrupole type, to approximately 1 Dalton in the case of narrowed quadrupole type, to 0.1 mDa or less when using FT-ICR, magnetic sector, or multi-turn TOF type. In general, the window width is set from several Daltons to 1 Da based on the trade-off with sensitivity. Under these analytical conditions, the product ion spectra can be a mix of isomers, isotopologes, isobars, polyatomic ions, product ions, and molecular ions of substances differing in the molecular mass by 1 or 2 Da.

Even if the sample is separated using LC, *etc.*, we must remember that there can be peaks from the solvent and the matrix, and from contamination with co-eluting substances. It may be possible to “remove” isobars, which are contaminants having different elemental composition, by using high-performance TOF mass analyzer, which has high mass resolving power and high mass accuracy for the product ion spectrum as well as applying the “mass defect filter” in an appropriate manner. This will be worth considering if the product ion spectrum is provided as a reference spectrum.

### 3.6 Using product ion spectra for calculation of reaction kinetic parameters of conversion of precursor ion

Calculation of the reaction kinetic parameters of the unimolecular dissociation reaction of precursor ion to product ion is possible by mass spectrometry. It requires two mass spectra. The one is a mass spectrum that analyzes the precursor ion without collision energy. This mass spectrum might observe the precursor ion alone or with isobaric contaminations. The other is a product ion spectrum measured under the analytical conditions specifying mass analyzer type, collisional energy level, collision gas molecule, gas pressure, *etc*. In these two measurements, the quantity of the precursor ion should be within the linear detection range of the detector. These two mass spectra as the reaction kinetics data make it possible to calculate how much of the precursor ion was converted to the product ions, and how much was lost by scattering and measurements outside of the range; this effect is particularly significant to the ion trap mass analyzer type. The reaction kinetic parameters calculated serve as an indicator of how “breakable” the precursor ion is and also proves useful for structure analysis.

Product ion spectra observing that 20–30% of the precursor ion remain intact are suitable for the calculation of the ratio of dissociated to intact precursor ions. However, spectra, measured applying too high or too low collision energy, where a significant amount of the precursor ion is dissociated or remains intact (∼90%), would be useful to structure analysis. In the case of using very low collision energy, it is possible to observe the product ions of higher *m*/*z* values that are helpful to give important insight into the structure of the substance.

### 3.7 Use of spectral libraries: Part 2

As the analytical conditions for the MS/MS of lipids are similar to each other, a spectral library intended for the identification of lipids has also been provided. This library contains many *in silico* product ion spectra predicted by considering the fact that these artificial spectra reflect the common patterns seen in lipid structures. However, it has become apparent that they lead to a larger number of misidentifications than expected.

### 3.8 Usefulness of product ion spectra measured under various analytical conditions

Until recently, MassBank was the only library available which had compiled product ion spectra resulting from MS/MS analysis of standard substances. As explained in Section 2.2, MS/MS analysis cannot reproduce any product ion spectrum unless the measurements are taken by faithfully reproducing the analytical conditions described in the metadata. Despite this, many libraries provide almost no information related to metadata. Furthermore, an overall representation of the structure of a given molecular ion can be obtained by measuring product ion spectra under various analytical conditions. If we take this into consideration, selecting one set of conditions out of many and treating that set as standard for the analytical conditions would limit the amount of structure information that can be obtained. Therefore, there is absolutely no need to standardize the analytical conditions.

Regardless of the analytical conditions under which a product ion spectrum is measured, a spectrum will form an important piece of experimental data containing the information related to the structure of the molecular ion as long as the spectrum is measured correctly and the metadata is recorded.

### 3.9 MS informatics tools

It is still technically impossible to perform structure analysis on the product ion spectrum of unknown substance for which we have no information about the structure. This is a serious limitation for structure estimation and MS informatics tools can help overcome this.

The CASMI contest (http://casmi-contest.org/) is a contest of MS informatics tools that elucidate the structure of unknown substances from their product ion spectra. By using the tools that have been successful in producing good results, it might be possible to elucidate the structure of a substance from its product ion spectrum. One can select a few of the elucidated structures and then check by structure analysis to make sure that the predictions match the observed product ion, and then choose the best candidate from among these.

### 3.10 Enriching “known information”

As proposed in these guidelines, we recommend submitting correctly measured spectra to MassBank and getting them published. Even when measuring the product ion spectrum of a substance with a known structure, a number of preliminary experiments are likely required in order to find the optimum measuring conditions for ionization and dissociation. As each of the many mass spectra obtained through such preliminary measurements gives the information on the product ions associated with the structure and physicochemical characteristics of the substance, it is recommended that these spectra are published from MassBank (Section 3.8). Every single spectrum is such valuable experimental data that become a source of existing knowledge about the relationship between bond cleavage and mass spectrum.

There is no harm in submitting the mass spectrum of a known substance to the MassBank, even if it is already published there. Data redundancy is important for the structure analysis of product ion spectra. Additionally, since only a few mass spectra measured by the EI-MS method on an instrument of high mass resolving power are deposited to MassBank, it would also be useful to publish them.

## Notes

Mass Spectrom (Tokyo) 2019; 8(1): A0076

## Notes

This article is a secondary publication of *J. Mass Spectrom. Soc. Jpn.* 66: 30−35, 2018 (Japanese).

## Notes

(1) In these guidelines, we will collectively refer to the ions that directly indicate the molecular mass, such as the molecular ion (M^+^*) and proton-added molecule ([M+H]^+^), *etc.*, as the “molecular ion.”

## Notes

(2) When you will find measured *m*/*z* values of peaks in a mass spectrum are largely out of the range of accuracy of the instrument. the instrument is incorrectly calibrated. In addition, other experimental steps, such as sample preparation and separation, may very likely be inadequate. Such spectra should therefore be discarded. It is recommended to prepare the sample again and to calibrate the instrument.

## Notes

(3) There are various MS methods as well as measuring conditions and purposes of the measurement. Metadata should be created to keep record of each of these situations using appropriate technical terms. The MassBank Database Committee provides advice on creating metadata and is also providing templates for this purpose. Please contact the committee by email (msdb@mssj.jp).

## Notes

(4) If M^+^* is not observed by EI-MS, the use of soft ionization methods such as chemical ionization (CI), fast atom bombardment (FAB), electrospray ionization (ESI), or matrix-assisted laser desorption ionization (MALDI) is recommended.

## Notes

(5) If you confirm that the sample and a standard substance are one and the same compound in terms of the stereochemistry, other analytical chemistry methods such as optical rotation should be used in combination with MS.

## Notes

(6) The degree to which the sample and the library mass spectra match is shown as a score. There is no relationship between the score and the probability that the structures of the two substances agree. There are many other factors to consider when using the library (Section 3.4).

